# Biological Rhythms in the Skin

**DOI:** 10.3390/ijms17060801

**Published:** 2016-05-24

**Authors:** Mary S. Matsui, Edward Pelle, Kelly Dong, Nadine Pernodet

**Affiliations:** 1 The Estee Lauder Companies; 125 Pinelawn Rd., Melville, NY 11747, USA; epelle@estee.com (E.P.); kdong@estee.com (K.D.); npernode@estee.com (N.P.); 2 Institute of Human Nutrition, College of Physicians & Surgeons, Columbia University, New York, NY 10032, USA; 3 Environmental Medicine, New York University School of Medicine, New York, NY 10016, USA; 4 Materials Science and Engineering, Stony Brook University, Stony Brook, NY 11794, USA

**Keywords:** circadian rhythm, clock genes and skin, oxidative stress, skin aging, trans-epidermal water loss, sebum, skin barrier, keratinocyte differentiation, glucocorticoids, Krüppel-like factor 9

## Abstract

Circadian rhythms, ≈24 h oscillations in behavior and physiology, are reflected in all cells of the body and function to optimize cellular functions and meet environmental challenges associated with the solar day. This multi-oscillatory network is entrained by the master pacemaker located in the suprachiasmatic nucleus (SCN) of the hypothalamus, which directs an organism’s rhythmic expression of physiological functions and behavior via a hierarchical system. This system has been highly conserved throughout evolution and uses transcriptional–translational autoregulatory loops. This master clock, following environmental cues, regulates an organism’s sleep pattern, body temperature, cardiac activity and blood pressure, hormone secretion, oxygen consumption and metabolic rate. Mammalian peripheral clocks and clock gene expression have recently been discovered and are present in all nucleated cells in our body. Like other essential organ of the body, the skin also has cycles that are informed by this master regulator. In addition, skin cells have peripheral clocks that can function autonomously. First described in 2000 for skin, this review summarizes some important aspects of a rapidly growing body of research in circadian and ultradian (an oscillation that repeats multiple times during a 24 h period) cutaneous rhythms, including clock mechanisms, functional manifestations, and stimuli that entrain or disrupt normal cycling. Some specific relationships between disrupted clock signaling and consequences to skin health are discussed in more depth in the other invited articles in this IJMS issue on Sleep, Circadian Rhythm and Skin.

## 1. Fundamental Concepts

There are a number of excellent descriptions of the oscillations and control mechanisms that allow all living organisms to exist on a planet characterized by not only day/night cycles, but also rhythmic fluctuations in temperature, humidity, food availability, and other environmental stresses [1,2,3,4,5]. Most physiologic oscillations occurring over time with a reproducible waveform have a 24 h periodicity, but additional periods of less than or greater than 24 h have also been reported.

**Figure 1:**
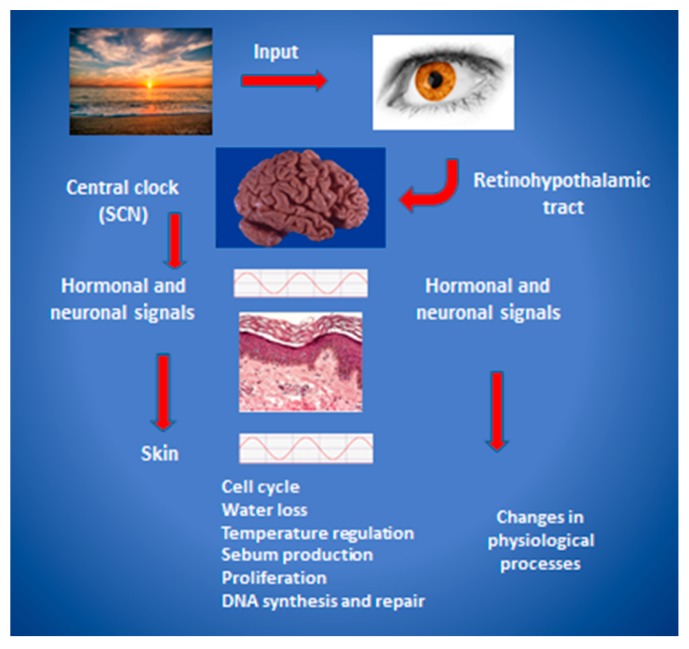
A model of hierarchical regulation and organization of the mammalian circadian clock. Light enters the retina and stimulates signaling via the retinohypothalamic tract to entrain the suprachiasmatic nucleus (SCN), located in the anterior hypothalamus. Hormonal and neuronal intermediaries then induce modulations of peripheral transcriptional and posttranslational responses that ultimately produce behavioral, metabolic, and physiologic output. The skin and its appendages are the focus in this figure, and key aspects of circadian regulation of physiological processes in the skin are listed. Modified from [7] with permission.

Visible light is considered the most powerful evolutionarily conserved organismal “master” clock entrainment cue in humans, acting through the retina (not directly on peripheral tissues). As shown in Figure 1, light acts to reset the central pacemaker located in the suprachiasmatic nucleus (SCN), which then initiates hormonal and neuronal signals that coordinate oscillations in physiological processes throughout the body. Peripheral organs and cells are functionally synchronized using additional cues such as nutritional components, through autonomous clocks driving oscillating expression of gene products and metabolites. For example, it was recently shown that feeding rhythms or adrenal hormones are required to synchronize clock gene rhythms in the liver with the SCN [6]. The impact of this system on human skin will be discussed here as it regards the periodicity of skin function and properties, the control of rhythms at a cell and molecular level, and therapeutic and clinical implications of circadian rhythm in skin health and disease.

**Figure 2:**
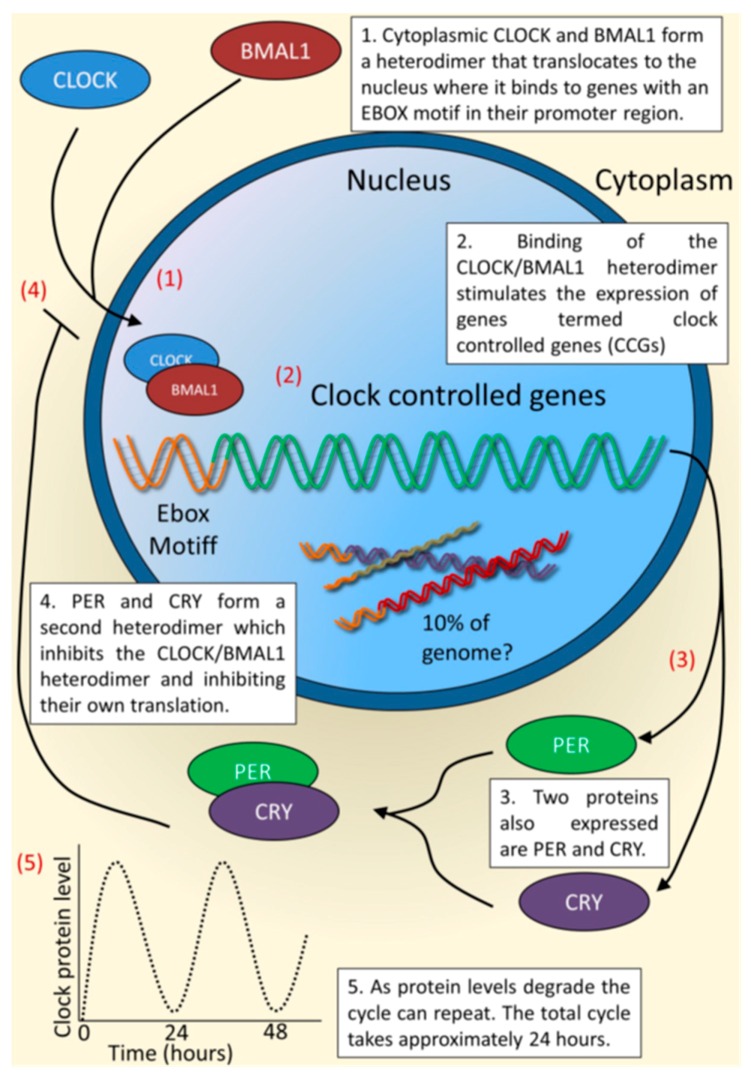
The core molecular clock system from Hardman *et al.* [23]. The figure shown describes a peripheral system in which the key elements in maintaining oscillations are shown. (**1**) The transcribed protein CLOCK and BMAL1 form a heterodimer, which translocates to the nucleus where it binds to genes containing an E-box motif in the promoter region or clock controlled genes (CCGs); (**2**) this binding leads to the transcription of CCGs and two families of proteins, the Period (PER) and Cryptochrome (CRY) family; (**3**) proteins PER and CRY form a second heterodimer, which prevents the formation of the first heterodimer and thereby inhibits their own transcription; (**4**) this cycle leads to an approximately 24 h oscillatory rhythm (**5**). Used with permission.

At a cellular level, the circadian clock mechanism is composed of interdependent feedback loops of transcription and translation of specific gene products, as shown in Figure 2. The dimer CLOCK/BMAL1 transcription factor drives expression of target genes such as Period 1–3 (*Per1/2/3*) and Cryptochrome 1 and 2 (*Cry1/2*) by binding to E-box elements in their promoters. Negative feedback response is provided by PER/CRY protein complexes that shuttle back into the nucleus, where they block CLOCK/BMAL1-mediated transactivation, thereby inhibiting their own transcription. In a second feedback loop, the orphan nuclear receptor REV-ERBα rhythmically represses Bmal1 transcription, presumably adding to the robustness of the circuitry. The master clock, through both neuronal signaling and neuroendocrine output, coordinates circadian expression of tissue-specific output genes and drives rhythmic expression of target genes [8]. The SCN integrates information from exogenous sources and under healthy conditions, synchronizes the downstream peripheral clocks. However, it is important to realize that skin cells, from keratinocytes, fibroblasts, melanocytes to mast cells and hair follicles, contain robust autonomous clocks [9,10,11]. As shown by high-resolution multi-organ expression data that nearly half of all genes in the mouse genome oscillate with circadian rhythm somewhere in the body. Transcriptional analysis seen in organisms from Neurospora, Drosophila, mice and human have revealed that up to 10% of gene expression in any tissue is rhythmical, as reviewed by Duffield [12]. In this manner, there exists tissue-specific circadian control of various physiological functions including cell growth, DNA damage-repair responses, metabolic processes or immune function. As a result, disruption of the circadian system can lead to pathological conditions such as metabolic syndrome, diabetes, and cancer (reviewed in [13]). This system is present is in nearly all cells, and is of primitive evolutionary derivation, elegantly described in reviews by Sassone-Corsi, Brown and others [5,14,15,16,17,18,19,20,21,22]. In the absence of external, entraining signals in excised tissue or cultured cells, central and peripheral expression of clock-controlled genes initially “free run” with a period of approximately 24 h followed by desynchronization due to the slight variations between individual cells (circadian means “about” 24).

## 2. The cutaneous Circadian System

For the most up-to-date and comprehensive discussion of the cutaneous circadian clock system, the reader is directed to Plikus *et al.*, who discuss a wide range of mechanistic research findings and their implications [24]. This 2015 review includes many aspects of the human skin for which there is insufficient space in this current review.

As pointed out by many authors, although the central master clock is entrained primarily by light entering the retina and stimulating the SCN via the retinalhypothalamic tract, the skin as an organ is in a unique position of interface between the environment and the organism and both receives and generates signals related to timing. Because the skin is exposed to the external environment, various functions, including the response to environment, are regulated and synchronized by centrally driven oscillations and can be reset locally by exposure to stimuli such as ultraviolet radiation (UVR), pollutants, humidity, and fluctuations in temperature. The impact of this on human skin will be discussed here as it regards the periodicity of skin function and properties, the control of rhythms at a cell and molecular level, and therapeutic and clinical implications of circadian rhythm in skin health and disease.

As shown in Figure 1, the master clock in the SCN communicates timing information to the skin via a combination sympathetic innervation and secreted hormones [7]. The skin as an organ is directly exposed to external conditions, including temperature, light, humidity, UV radiation and pathogens. Under normal, healthy conditions, it has been reported that many attributes of human skin follow a periodicity: hydration and transepidermal water loss (TEWL), capillary blood flow, sebum production, temperature, surface pH, keratinocyte proliferation rates, and even the visibility of facial rhytides [25,26,27,28]. Most likely due to the more permeable barrier at night, itch and irritation have also been reported to have both a circadian and an ultradian rhythm [29,30]. Luber *et al.* [28] have reviewed much of the published data describing the daily rhythmic patterns of the behaviors listed above. It should be noted that historically, experimental data have been obtained using non-human nocturnal animal or tissue culture model systems, which must be interpreted in light of a nighttime active phase. Other confounding issues are encountered with studies using cell cultures. For those reasons, in addition to others, data obtained from human clinical studies are emphasized in this review, particularly those studies that have attempted to control for as many variables as possible.

## 3. Clinical Phenomena of Cutaneous Rhythmicity

It has been reported that skin blood flow has a pattern characterized by low morning rates, with the highest rates in the afternoon and a second peak in the late evening just before sleep. Sleep is associated with decreased heat production and increased heat loss, the latter resulting from an increase in skin blood flow and skin temperature [31]. In fact, changes in perfusion and peripheral skin temperature may be functionally linked to sleep onset [32]. Skin temperature typically reflects cutaneous blood flow, with the lowest temperatures occurring in early morning and highest in the early evening. This correlates with an increased barrier permeability in the evening, and a higher incidence of itch [33].

In studies from 1970 to 1993, it was reported that the rate of facial sebum secretion varies with a circadian rhythmicity, lowest during the night and peaking in the early afternoon [34,35]. In a later study of eight synchronized subjects under controlled environmental conditions, ultradian and circadian rhythms were detected in facial (but not forearm) sebum secretion, with periods of 8 h in addition to the 24 h oscillation [25]. Interestingly, attempts to match the sebum excretion changes with free testosterone, dehydroepiandrosterone sulfate, Δ^4^-androstenedione, cortisol, or melatonin blood levels have been unsuccessful, which may imply that autonomous clocks in sebaceous glands are responding to other entrainment stimuli.

The study by Le Fur *et al.*, noted above, is of particular interest [25] even though the number of subjects was small. The investigators took care to control for light, temperature and humidity during a 48 h length of time. Meals and physical activity were strictly controlled. Measurements were taken on recumbent subjects every 4 h on fixed predetermined sites of the face and the volar forearm, their forearms in a horizontal position. In addition to sebum measurements, skin capacitance, skin temperature, transepidermal water loss, and skin surface pH were also monitored. Although there were no temperature fluctuations on the cheek site, two peaks in forearm skin temperature were recorded 12 h apart with a nadir in the middle, at noon. Capacitance, a measure of skin hydration, was too variable between days for a pattern to be discerned on the face, but did display an ultradian rhythm on the forearm. TEWL in this study and others has been found to have rhythmic values both on the cheeks and forearm, but the patterns have differed between studies [27,36,37,38]. A review of this and other reports indicates that study experimental parameters (including, diet, sampling frequency, relative humidity, and body site) and possibly other factors such as age, ethnicity, gender, and weight may influence observed rhythmicity—period length, phase, and amplitude [27,34,35,36,38].

## 4. Regulatory Factors in Cutaneous Rhythms

Whether environmental temperature change functions as a modulator of peripheral clocks and gene expression in the skin apart from time of day is unclear. At the cellular level, Spörl *et al.* in 2011 [39] were able to show a functional, cell autonomous, circadian clock in human (HaCaT) keratinocytes and were able to use temperature cycles to induce circadian transcription of canonical clock genes as well as several genes involved in skin barrier function, including cholesterol homeostasis and differentiation. It should be noted, however, that this observation about temperature as a regulatory factor has not been replicated in normal human keratinocytes or *in vivo*. In fact, these observations contrast with early findings by Denda and Tsuchiya that showed barrier repair to be time-dependent but not well correlated with temperature [40]. In addition, Le Fur *et al.* [25] reported that a rhythm of cheek TEWL was detectable in the absence of significant changes in skin temperature and concluded that skin temperature is not a primary regulatory factor for circadian variations of TEWL.

It is clear that all the major cell types in human skin have functional circadian machinery and display specific periods and phase relationships in gene expression, suggesting regulatory mechanisms that are particular to each cell type [41]. Furthermore, these oscillations in different cell types appear to act in concert to drive rhythmic functions in the skin. One limitation for the study of clock gene expression in human skin has been the need to biopsy living skin in subjects. Akashi *et al.* has now described a non-invasive method using hair follicle cells from plucked hair to monitor gene expression over time, and used this to show that rotating shift workers suffer from serious perturbations in their circadian gene expression [42]. In another highly detailed study of numerous pathways in epidermal stem cell differentiation, researchers found that oscillation of the core clock transcriptional machinery consists of successive waves during a 24 h day, during which keratinocytes respond in a time of day manner to differentiation cues [43]. The authors describe 4–5 h phase shifts that appear to provide functional landmarks that separate vital functions of keratinocytes, including proliferation, DNA repair, and differentiation.

Other observers have noted skin physiological parameters to follow time of day rhythms. TEWL is significantly higher in the afternoon and evening compared to the morning, suggesting that epidermal barrier function is less optimal late in the day and in the evening [25,27,30,36,38]. It has been suggested that the evening elevation of cutaneous blood flow, combined with higher evening TEWL levels, may contribute to the pruritus that eczema patients experience at night [28]. Twenty-four, 12- and 8-h rhythms were found by Le Fur *et al.* [25] for TEWL on the face, and 8-h periods for capacitance on the forearm. In general, research groups have found higher TEWL in the late afternoon and evening; however, results vary, most likely due to differences in study design, including the number of sampling times and location of sites and in environmental control. Although time of day-dependent variations in skin barrier recovery after tape stripping have been reported, it is not entirely clear what the ultimate molecular mechanisms for this are [27,30]. Experimental models have yielded much data that support the correlation between components of the clock and skin health and function, and are reviewed in detail elsewhere [19]. For example, related to behavior important to wound repair, disruption of circadian gating by eliminating PERIOD clock repressor proteins (per1/per2 mut lacking both proteins) resulted in fibroblast and keratinocyte hyperproliferation, and subsequent collagen down-regulation. Eliminating the BMAL1 clock activator protein resulted in suppression of cell proliferation and an increase in collagen secretion [44].

## 5. Rhythms in Keratinocyte Differentiation and Proliferation

Epidermal keratinocytes arise from the stem cell compartment, pass through the transient amplifying cell phase, and go on to proliferate and finally to terminally differentiate. Because they form the basis of a continuously renewing tissue, they have several levels of circadian, infradian (having an oscillation frequency of more than one day, such as monthly or seasonally) and ultradian regulation over a lifespan of roughly two months, all the while responding to both systemic and environmental cues.

**Figure 3:**
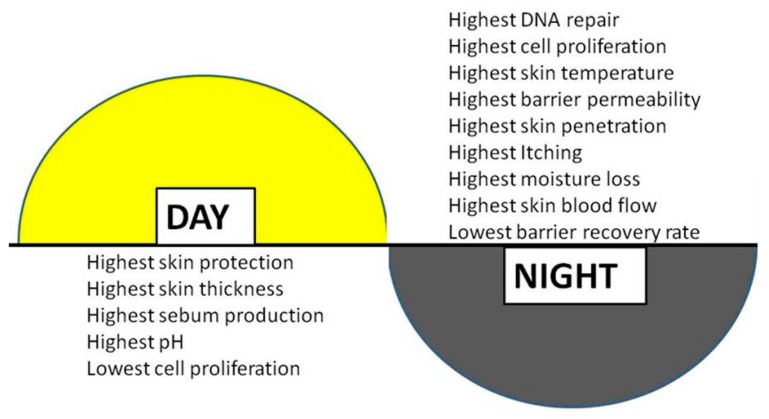
The oscillation of human skin properties as they cycle through the day and night. For example, sebum production has been shown to be higher during the daytime that at night. Higher values for itch and moisture loss occur during the evening. Adapted from, *Harry’s Cosmeticology 9th Edition*, Pernodet and Pelle with permission [47].

One of the primary chronobiological functions of the interfollicular epidermal clock, as described in Plikus *et al.* [24], is to regulate cell differentiation and proliferation for optimal function, and this certainly applies to the skin, allowing it to respond to predictable changes in temperature, humidity, UVR, and other environmental stressors. In human epidermis, cellular proliferation in keratinocytes has been measured to be 30-fold higher at night than at noon [45] and epidermal stem cells show a similar pattern and have a higher rate of proliferation at night *versus* day [43]. The epidermis undergoes a constant process of self-renewal in which epidermal stem cells commit to a complex program of terminal differentiation. Precise spatial and temporal control of keratinocyte proliferation and cell cycle withdrawal is critical to this phenomenon [46]. Skin cell division, as well as DNA replication and repair, have long been observed to occur with high correlation to a diurnal type cycle and the clinical implications of this are significant. These rhythms impact both acute (erythema, DNA damage and immune suppression) and long-term (skin cancers and photo aging) consequences of UVR exposure. . Skin responses to short and long wave ultraviolet radiation (UV), Ultraviolet B (UVB) and Ultraviolet A (UVA) exposure, are under the influence of circadian rhythms via a complex relationship with the intrinsic master clock-directed circadian oscillations in DNA repair capacity, which is then reflected in a circadian erythemal response to sun exposure. Interestingly, it has been suggested that the circadian clock functions in epidermal keratinocytes to temporally segregate endogenous oxidative phosphorylation from keratinocyte proliferation, thus protecting the genome from endogenous reactive oxygen species (ROS)-mediated DNA damage [9]. This may result in a high vulnerability in humans to UV-induced skin cancers. In addition, induction of DNA repair enzymes by UVR-induced DNA lesions occurs with a magnitude reflective of the time of day. The yin-yang nature of timing for optimal DNA repair *versus* cell proliferation, with the circadian rhythmicity of DNA replication anti-phase to that of DNA repair, is well established and is indicated in Figure 3 [47]. Additionally, DNA lesions such as *O*^6^-alkyl-guanine and 8-oxo-dG have been shown to be repaired at higher rate during the night [48,49].

## 6. Circadian Influence on DNA Repair, Skin Cancer, and Skin Disease

That a circadian influence exists for the risk of UVR-induced mutation and subsequent skin cancer has been shown by several groups and excellently reviewed recently [50], although much of the data has been obtained from murine models. Sensitivity to UVB-induced DNA damage in mouse epidermis is time-of-day and BMAL1 dependent. UV sensitivity is higher during the peak phase of DNA synthesis, the cell-cycle stage most vulnerable to DNA damage [9]. Sunburn-induced apoptosis, induction of inflammatory cytokines, and erythema were all shown to have circadian fluctuations, with mice more susceptible to skin cancer induction following chronic irradiation in the morning when compared with evening irradiation [50]. Any alteration to this fine-tuned mechanism can have dramatic consequences. In 2013, Lengyel *et al.* [51] presented the first clinical evidence that there might be a direct link between circadian clock genes and human skin tumorigenesis. By comparing biopsies from human melanoma to non-tumorous samples, they were able to show that the expression of *Per1*, *Per2*, Clock and *Cry1* clock genes and corresponding protein levels in the nucleus were reduced by 30%–60% in melanoma *versus* normal skin. As well, Kuaskoff and Weinstein hypothesized that the increased incidences of melanoma in recent years observed indoors and in office workers to fluorescent light might be due to exposure to artificial light at night as it promotes melatonin suppression [52]. In addition, a study by Li *et al.* [53] correlating the increased incidence of psoriasis in shift workers suggests that their circadian disruption could be more important for skin diseases and disorders than previously recognized. A mechanistic link between psoriasis and the circadian clock was suggested by a study in mice which provided evidence that Clock may regulate psoriasis-like skin inflammation in mice via direct modulation of IL-23R expression in γ/δ + T cells [54].

Circulating levels of glucocorticoids are one of the main mechanisms by which the master clock, the SCN, regulates and synchronizes peripheral tissues. This regulatory pathway uses glucocorticoids to transduce photic signals to peripheral tissues, which are not intrinsically photo-responsive [55]. Glucocorticoid receptors mediate circadian clock entrainment in peripheral tissues, while the presence of glucocorticoid response elements (GREs) in several clock genes, including *Per1/2/3*, suggests their involvement at the level of expression [56]. Oscillation patterns may alter the efficacy and toxicity of topical medications and products because xenobiotic metabolism and circulating glucocorticoid levels fluctuate in a time related manner (for review, see reference [55]).

## 7. Epidermal Nuclear Receptors and Transcription Factors

The importance of nuclear receptors (NRs) cannot be overstated with regard to bi-directional communication between the master clock and the peripheral organs, and has been recently reviewed [55]. With regard to skin homeostasis and adaptive modulation, NRs are critical, and can be regarded as having exciting new potential as therapeutic targets involving modulation of clock-controlled genes. A specific example includes the peroxisome proliferator-activated receptor (PPARa), a CLOCK- and BMAL1-regulated gene, owing to the presence of an E-box in its promoter. Circadian variation in lipid metabolites appears to be regulated to PPARs, PPARc and PPARd. NRs pregnane X receptor (PXR) and constitutive androstane receptor (CAR) are thought responsible for circadian regulation of xenobiotic metabolism. For a number of reasons, NRs are currently receiving considerable attention for their promise as pharmaco-therapeutic targets.

An excellent example of communication between the master clock and skin is a key epidermal circadian transcription factor regulating keratinocyte proliferation, Krüppel-like factor 9 (Klf9), which appears to have rhythmic expression driven by systemic cortisol and possibly also temperature [26]. This work on Klf9 is a valuable contribution to the study of skin circadian rhythm, utilizing repeated sampling and genome-wide transcriptional profiling for time dependent variations in gene expression. Klf9 expression is highly sensitive to glucocorticoids and in human subjects shows diurnal expression patterns in phase to systemic cortisol rhythms. Substantial inter-individual variability was detected in the amplitudes of daytime-dependent genes, similar to that seen in other studies, and probably attributable to inter-individual differences in the epidermal oscillator. This type of work will be of great importance in understanding the impact of circadian disruption on the etiology of many skin disorders from premature skin aging, fluctuations in the response to UVR, diurnal exacerbations of psoriasis and atopic dermatitis, to the risk for and modulation of skin cancer.

There is accumulating evidence that dysregulation of the clock mechanisms leads to increased generation and accumulation of ROS and oxidative stress [57]. In fact, near lethal oxidative stress has been reported to induce resetting of the clock and synchronization in cultured murine fibroblasts [18]. The ROS-triggered clock resetting and pro-survival responses were mediated by transcription factor, central clock-regulatory BMAL1 and heat shock stress-responsive (HSR) HSF1. The secretion of an endogenous antioxidant, melatonin, is regulated by the circadian clock and it has been stated that dysregulated circadian control of melatonin can contribute to the adverse effects of UVR on the skin [3]. Aging itself is known to result in increased oxidative stress in most organs and tissues, but the exact mode of action of this involving the circadian clocks is not fully clear, nor is the “cause and effect” relationship.

**Figure 4:**
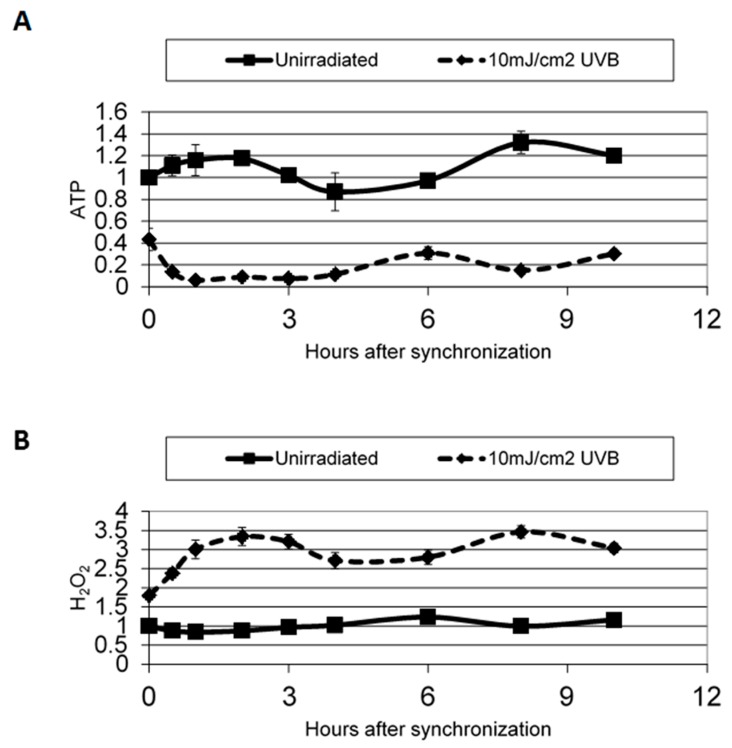
ATP (**A**) and H_2_O_2_ (**B**) levels after release from starvation in irradiated and non-irradiated cells. ATP and H_2_O_2_ levels were measured at several time points after being released from starvation. ATP and H_2_O_2_ levels are shown as relative to the level at 0 h. ATP and H_2_O_2_ rhythmicity is lost after UV irradiation. Error bars are standard error measurements. Used with permission, Experimental Dermatology, Dong *et al*. [60].

Since Kawara *et al.* [58] reported a disruption in clock gene expression in normal human epidermal keratinocytes after UVB irradiation (10 mJ/cm^2^) and because it is well-known that UVB can induce reactive oxygen species [59], we investigated the effects of environmental trauma in skin cells as a function of time. Our results showed a temporal pattern in hydrogen peroxide (H_2_O_2_) generation, as well as a disruption in ATP synthesis in synchronized cells after UVB exposure (Figure 4), and support the connection between clock gene activity and oxidative stress. This intimate link is critical for controlling skin aging [9,59].

## 8. Aging and Cutaneous Rhythms: Circadian and Ultradian

Aging itself is known to result in increased oxidative stress in most organs and tissues, and a relationship with dysfunction of circadian clocks has been reviewed in detail elsewhere [61]. The authors discuss research that shows (a) a correlation with aging and circadian disruption; (b) that mutations in clock genes shorten life span; and (c) that longevity can be extended by transplantation of fetal clocks into the brains of aged animals. Clock genes play an important role in maintaining homeostasis and responding to oxidative stress.

Recent evidence indicates that chronological aging leads to a diminution in the function of the master zeitgeber, with myriad consequences to optimal functioning, sleep and wake states, and susceptibility to pathology. Degradation of the clock signals is a major contributor to changes in hair growth and pigmentation [62], and also contributes to chronic wounds in the elderly [63]. Conversely, a decline in the robustness of circadian rhythms due to the passage of time or societal and behavioral disregard for the “natural” sleep/wake rhythm or proper nutrition content and timing may contribute to the aging process. It has been suggested, for example, that circadian genes may play a role in aging-related alopecia, which is characterized by aberrations in the hair growth cycle [62]. Clock genes have been shown to modulate human hair follicle cycling and as an integral component of the human hair cycle clock, BMAL1 and Period1 are viewed as potential targets for modulating hair growth [10]. Other work describing the impact of chronic short sleep certainly suggests that disruption of circadian rhythm is correlated with signs of accelerated skin aging and diminished skin barrier function [64].

**Figure 5:**
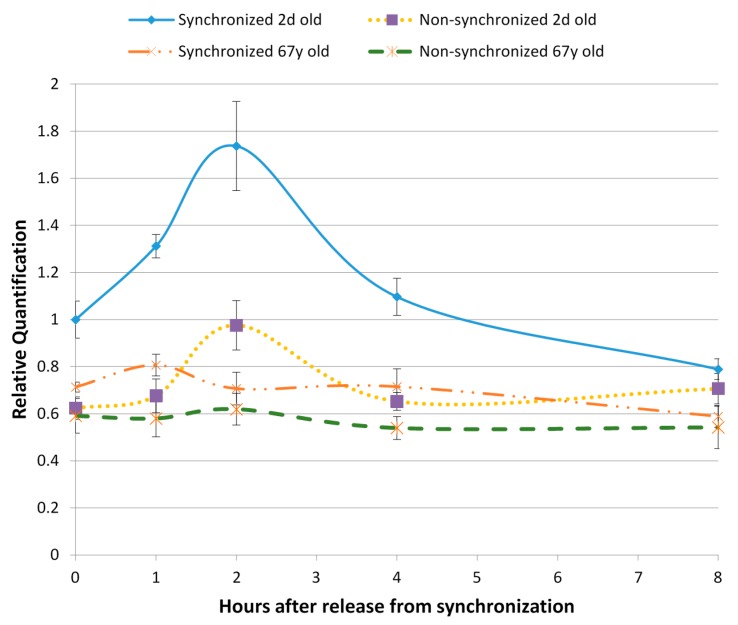

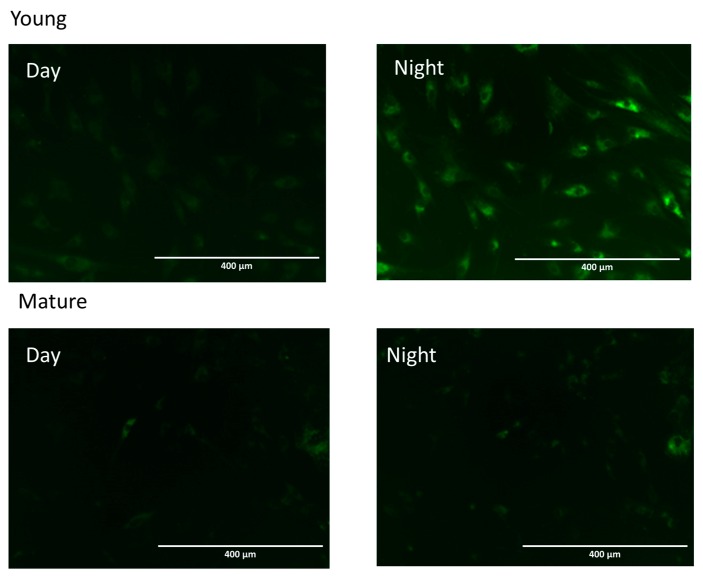
Cell synchronization was induced by nutrient deprivation followed by nutrient repletion, which induced a temporal rhythm that showed an increase of autophagy LC3B by 77.9% in young fibroblasts, whereas this effect was absent in aged fibroblasts. LC3B expression was determined by RT-PCR and normalized to glyceraldehyde 3-phosphate dehydrogenase (GAPDH) housekeeping genes (*n* = 3). In addition, temporal rhythm was followed over a 24 h period through fluorescent imaging specific to LC3B, showing the higher fluorescence at night *versus* day in young human fibroblasts but lost in aging human fibroblasts. Used with permission, *Journal of Cosmetic Science*, Pernodet *et al.* [65].

Our work describing the impact of chronic short sleep certainly suggests that disruption of circadian rhythm is correlated with signs of accelerated skin aging and diminished skin barrier function [64]. At the cellular level, we studied the impact of aging on normal human dermal fibroblasts by following over time one specific and critical mechanism: autophagy [65]. Under synchronized conditions, young human fibroblasts showed a clear temporal rhythm in microtubule-associated proteins 1A/1B light chain 3B (LC3B), a biomarker for autophagy, as demonstrated by reverse transcription polymerase chain reaction (RT-PCR) and specific fluorescent staining (Figure 5). The LC3B peak was absent in aging fibroblasts, even though they were synchronized, indicating not only a loss of temporal rhythm but also a loss of autophagy, which would have significant consequences in accelerating aging. This has also been reported by Ma *et al.* [66], showing the temporal orchestration of circadian autophagy rhythm.

There is accumulating evidence that dysregulation of the clock mechanisms leads to increased generation and accumulation of reactive oxygen species (ROS) and oxidative stress [34]. In fact, near lethal oxidative stress has been reported to induce resetting of the clock and synchronization in cultured murine fibroblasts [14]. The ROS-triggered clock resetting and pro-survival responses were mediated by transcription factor, central clock-regulatory BMAL1 and heat shock stress-responsive (HSR) HSF1. More detailed information at a single cell level about the relationship between cellular rhythms and metabolic oscillations has been recently examined using a non-invasive method, two-photon excitation and fluorescence lifetime imaging microscopy in live mice [67]. The researchers used the intrinsic metabolic biomarker NADH to detect metabolic oscillations and circadian phase within epidermal stem cells and observed a higher NADH/NAD+ ratio, indicating an increased glycolysis/oxidative phosphorylation ratio during the night compared to the day. As with the work cited in the paragraph above, dysregulation of the circadian clock leads to increased generation of ROS, since the circadian clock confers time-of-day dependent shifts in glycolysis *versus* oxidative phosphorylation within proliferating epithelial stem cells, thus minimizing DNA damage during S-phase [67].

The secretion of an endogenous antioxidant, melatonin, is regulated by the circadian clock and it has been stated that dysregulated circadian control of melatonin can contribute to the adverse effects of UVR on the skin [3]. Perhaps not surprisingly, it was found that all three major cell types in the skin, keratinocytes, dermal fibroblasts and melanocytes metabolize melatonin, and melatonin and its metabolites play a role in building and maintaining the epidermal barrier [68].

## 9. Conclusions and Implications for Future Study

Research reports, reviews and discussions of many other aspects of human circadian clocks can be found in the scientific literature but will not be reviewed here, including the relationship with pigmentation [23,69], stem cell differentiation [62], pharmacokinetics and toxicology [70,71,72], and hair growth [19,23,61,73]. Metabolomic analysis of the circadian rhythm has also recently begun and marks the beginning of a deeper understanding into the complicated network of clock signaling [22].

In summary, circadian rhythms and their impact on human life is no longer simply a curiosity, but rather should inform behavior and therapy. There are currently almost 70,000 references listed in PubMed for the key words “circadian rhythm”, beginning with one paper in 1945, double digits per year until about 1965, rising to just under 1000 until 1984 and now topping 2000/year. The skin is clearly characterized by rhythmicity in virtually all its functions, which argues in favor of the recommendation that individual rhythms be taken into account for diagnosis and treatment schedules. Encouragement should be given to the discovery of new treatment targets based on clock signaling, targeted bioactive agents, and the development of strategies to maintain a healthy periodicity through appropriate sleep/wake cycles, diet, exercise, and other behaviors. As discussed elsewhere in this IJMS issue on Sleep, Circadian Rhythm and Skin, there appear to be serious consequences for disrupting this embedded behavior. It seems wise to take into account the fact that we have evolved on a planet that, for billions of years, has been rotating on its axis, leading to the rhythmic repetition of days and nights. For an excellent review of the past, present, and future of research in this topic, see: *The Time of Your Life* by Sassone-Corsi [5].

## Abbreviations

TEWL	Trans-epidermal water loss
Klf-9	Krüppel like factor 9
SCN	Suprachiasmatic nucleus
CCGs	Clock controlled genes
PER	Period
CRY	Cryptochrome
GREs	Glucocorticoid response elements
NRs	Nuclear receptors
ATP	Adenosine Triphosphate
ROS	Reactive oxygen species
UV	Ultraviolet
UVR	Ultraviolet radiation
UVB	Ultraviolet B
